# Prenatal chlorpyrifos leads to autism-like deficits in C57Bl6/J mice

**DOI:** 10.1186/s12940-017-0251-3

**Published:** 2017-05-02

**Authors:** Anat Lan, Michal Kalimian, Benjamin Amram, Ora Kofman

**Affiliations:** 1 Department of Psychology, Ben-Gurion University of the Negev, P.O.B. 653, Beer-Sheva, 84105 Israel; 2 Zlotowski Centre for Neuroscience, Ben-Gurion University of the Negev, Beer-Sheva, Israel

**Keywords:** Autism, Chlorpyrifos, Pesticide, Prenatal, Social deficit, Restricted interest

## Abstract

**Background:**

Children are at daily risk for exposure to organophosphate insecticides, of which the most common is chlorpyrifos (CPF). Exposure of pregnant women to CPF was linked to decreased birth weight, abnormal reflexes, reduction in IQ, as well as increased maternal reports of signs of pervasive developmental disorder. The aim of current study was to examine the long term effects of prenatal exposure to CPF in C57BL/6 J (B6) mice with specific focus on social and repetitive behavior.

**Methods:**

B6 female mice were treated with vehicle, 2.5 mg/kg CPF or 5 mg/kg of CPF on gestational days 12–15 by oral gavage. On postnatal days (PND’s) 6–12 early development and neuromotor ability were assessed by measuring 3 neonatal reflexes in the offspring. In adulthood, PND 90, social behavior was investigated using the social preference, social novelty and social conditioned place preference tasks. Object recognition and restricted interest, measured by the repetitive novel object contact task (RNOC), were also assessed on PN D 90. In order to rule out the possibility that CPF administration induced alterations in maternal care, the dams’ behavior was evaluated via the maternal retrieval task.

**Results:**

CPF treatment resulted in delayed development of neonatal reflexes on PND’s 6–12. On PND 90, mice treated prenatally with the 5.0 mg/kg dose exhibited reduced preference towards an unfamiliar conspecific in the social preference test and reduced social conditioned place preference. In the RNOC task, mice exposed prenatally to 2.5 mg/kg dose of CPF showed enhanced restricted interest. CPF administration did not impair dams’ behavior and did not cause memory or recognition deficit as was observed in the object recognition task.

**Conclusions:**

Our data indicate that gestational exposure to CPF has long-term deleterious effects on social behavior and limits exploration of novel objects.

## Background

Chlorpyrifos (CPF) is one of the most widely-used organophosphate pesticides (OP), for the control of insects in both agriculture and urban communities [1]. Indoor use of CPF was phased out in 2005 in the US [2], and the primary route of human exposure is via ingestion of foods containing CPF residues [3, 4]. In agricultural communities, exposure pathways also include dermal contact and inhalation [5]. Young children, compared to adults, are more susceptible to acute toxicity of CPF [6, 7], and this could be explained, in part, by the fact that immature animals have lower levels and activity of enzymes that hydrolyze CPF [8, 9].

CPF targets the central and peripheral nervous system, acting primarily by inhibiting acetylcholinesterase (AChE), resulting in hyper-stimulation of acetylcholine (ACh). Exposure to CPF was also found to elicit changes in cholinergic and catecholaminergic markers in the brain [10], and transcription factors involved in neural cell replication and differentiation [11], as well as stunted axonal growth [12], suggesting that CPF has non-cholinergic mechanisms of action, as well. In subtoxic doses, that do not cause overt signs of toxicity, CPF caused significant inhibition of DNA and protein synthesis, as well as decreased cell density in forebrain, cerebellum and brainstem of neonatal rats [13]. Subtoxic exposure to CPF was also found to affect the expression levels of critical genes involved in fetal brain development, including genes involved in motor abilities, learning [14], neuronal communication, growth and plasticity [15].

The neurotoxic effects and neurodevelopmental outcomes of subtoxic exposure to CPF in humans were evaluated in several studies, which found associations between prenatal exposure and developmental deficits in infancy and childhood. Gestational OP exposure was associated with 1) a reduction in infant body length and weight [16], 2) significantly reduced scores in the Mental Development Index (MDI) at ages 12 and 24 months [17], 3) parental reports of Pervasive Developmental Disorder (PDD) at age 24 months [18], 4) lower scores on the Psychomotor Development Index at 36 months [19], and 5) attention deficits at age 36 months [19] and 5 years [20]. Moreover, in the ongoing longitudinal studies, gestational CPF was associated with lower IQ scores and impaired working memory at about 7 years of age [17, 21, 22], a deficit that was found mainly in boys [23]. In 7–9 year old children, gestational exposure was associated with impaired social skills in black participants and in boys [24].

These studies establish OPs as a long-term risk factor for developmental disorders, but due to lack of statistical power, prospective studies of gestational exposure in infants are limited in their ability to examine disorders like Autism Spectrum Disorder (ASD) [25]. Studies in laboratory animals can demonstrate the causative relationship between an early exposure to CPF and neurodevelopmental impairments. Another advantage to assessing gestational exposure in laboratory animals is that often epidemiological studies are confounded by differences in rearing, nutrition and living conditions amongst mothers who have high vs low levels of OP metabolites in their urine, which can result from greater consumption of vegetables and fruit [26]. A higher level of OP metabolites in mothers was associated with higher socioeconomic status, ethnicity, and education and better diets, factors which can affect the developmental behavioral measures [27, 28].

Administration of CPF to rodents induced long-term behavioral abnormalities, namely deficits in the righting and geotaxis reflexes in female rat pups and reduced locomotor activity in male rat pups after postnatal exposure to CPF on PND 1–4 [29], enhanced agonistic behavior when encountered with an unfamiliar conspecific in male mice after CPF treatment on GND 15–18 and PND 11–14, and decreased anxiety responses on the plus-maze test in mice exposed to CPF on PND 11–14 [30]. CPF exposure elicited decreased ultrasonic vocalizations (USV) in mouse pups, suggesting an impaired ability to communicate distress to the dam [31], an effect that was also found in male and female mice exposed during gestation to chlorpyrifos oxon [32]. However, this last study did not find long term effects on social preference or social novelty compared to vehicle treated mice. Although these studies suggest isolated signs of ASD-like developmental disorders, they have not been robustly replicated in different studies, possibly due to methodological differences between labs.

The aim of the current study was to test the hypothesis that gestational CPF impairs development in several different domains, including motor development and social behavior. C57BL/6 J (B6) mice were treated with vehicle, 2.5 mg/kg or 5 mg/kg of CPF during gestational days 12–15, a period which corresponds roughly to the second trimester of pregnancy in humans [33]. The doses used in the current study were similar to those used in studies on gestational exposure of CPF in rodents. Previous studies found that 3 or 6 mg/kg gestational CPF from gestational days 15–18 inhibited serum, but not brain AChE [34, 35] and a similar treatment regimen (5 mg/kg gavage on GD 6–10) elicited significant cholinesterase inhibition on PND 1 and 5 in the brain and a more prolonged inhibition in blood and heart [36]. The highest dose of CPF used in the current study was found to elicit 20% inhibition of AChE in mice exposed during late gestation and postnatally [37].

Exposure to CPF in humans is unintentional, but has been found to be widespread in American [38, 39] and Asian children [40]. Exposure in children occurs via different means such as dust, inhalation, dermal exposure and food and includes a mixture of OP and substances [41]. Hence, investigating teratogenic effects of controlled gestational exposure to CPF enabled us to overcome some of the variability that confounds teratogenic research in humans, with the intention of providing a solid basis for exploring the mechanism of the reported deficits in future research.

In the current study, only male offspring were tested, as males are at higher risk for ASD [42]; however, a study on both sexes is underway in our laboratory. To evaluate pups’ early development and neuromotor ability, we tested 3 neonatal reflexes; the righting reflex, negative geotaxis, and cliff avoidance. Social behavior was evaluated in adulthood by tests for Social Preference (SP), preference for a conspecific over an object, Social Novelty (SN), preference for a novel mouse over a familiar mouse and Social Conditioned Place Preference (SCPP), preference for an environment previously conditioned with a social stimulus rather than an environment that was not conditioned with such stimulus. B6 mice have been found to display all three of the above signs of social behavior [43–45]. Repetitive behavior was tested using the Repetitive Novel Object Contact task (RNOC) which was used in the proprionic acid model for autism [46]. Finally, in order to rule out the possibility that the maternal behavior of the treated dams was impaired, we examined dams’ maternal behavior.

## Methods

### Animals and treatment

All experiments were conducted on mice of the B6 inbred strain, using only one mouse per litter. Dams and sires for breeding were purchased from Harlan, Israel. Except for the maternal behavior task, all tests were done on male mice. Animals were maintained in a temperature controlled environment (22 ± 1 °C) under a 12-h reversed light-dark cycle (21:00–9:00 lights on) and *ad libitum* food and water. Reflexes were tested on PND 6–12 and behavior in adults was tested on PND 90. The protocols were approved by the Institutional Committee for the Ethical Care and Use of the Animals in Experimentation of Ben-Gurion University of the Negev.

CPF (99.5% purity, Chem Service, Inc.) or corn oil (Willi Food, Yavneh, Israel) was administered by gavage to pregnant females daily from GND 12 to 15 in a volume of 0.1 ml/10 g body weight using a 22 gauge stainless steel feeding tube (Solomon Instech, Inc.). The gestation of B6 mice is 462.4 h long, or 19.2 days [47], slightly shorter than that observed in CD-1 mice used in other labs. This period is equivalent to Theiler stages 23–26 of development [48], and more specifically to days 61–84 of human brain cortical development and 50–67 of human brain limbic system development [49].

In the first series of studies, 29 dams were divided into 4 treatment groups: No Treatment, Vehicle (corn oil), 2.5 or 5 mg/kg CPF. Only one male from each litter was used for each experiment, and no animal was used in more than one experiment, such that the adults in the SP and RNOC studies were siblings of the pups used in the reflex study. The SCPP study was done on a separate cohort of 32 dams, divided into 4 groups, as above. The stimulus mouse housed with the experimental mouse was taken from the same litter, in order to control for familiarity, but was not tested for bedding preference. The maternal care study was a pilot study done on a separate cohort of four female mice per group.

### Maternal care/Dams’ behavior (PND 5)

In order to test whether maternal care was impaired by CPF, on PND 5 dams were assessed in the maternal retrieval task. Pups were isolated from their dam, which was left alone in the home cage for 15 min. The pups were kept warm during this interval by placing them on a towel wrapped hot water bottle at 37 °C. Three male pups from the litter were returned to the home cage and placed fixed areas about 8 cm from the nest. The latency to retrieve all three pups to the nest was scored by an experimenter blind to the treatment.

### Assessment of early development and neuromotor behavior (PNDs 6–12)

To evaluate pups reflex development and neuromotor ability, 3 neonatal reflexes were assessed blindly from PND 6–12 in male pups: The righting reflex, negative geotaxis and cliff avoidance. The data represent the average of 3 trials performed sequentially, at intervals of 1 min. The maximum time allowed per trial was 30 s. The pup was weighed before testing.

#### Righting reflex

In this test, each mouse was placed on its back on a flat surface and released. The amount of time required to turn upright with all 4 paws in contact with the surface was recorded on a stopwatch.

#### Negative geotaxis reflex

Pups were placed in head-down position on a 25° inclined surface. They had to complete a 180° turn and maintain the position for 30 s. Mouse performance was manually ranked on a 1–3 scale: 1) downwards slide, 2) remained in position without turning, 3) 180° turn within 30 s.

#### Cliff avoidance reflex

Each animal was placed on a table edge with the forepaws at the edge and the whiskers and nose extending over the edge. To successfully perform this reflex, the pup had to turn 180° and turn away from the edge within 30 s. Mouse performance was manually ranked on a 1–4 scale: 1) fell on soft padded surface, 2) remained in position without turning, 3) partial turning, 4) 180° turn.

### Social behavior in adults (PND 90)

#### Social preference (SP) and social novelty (SN)

In the SP task a mouse was placed in a 24 X 16 cm three chambered box in which the two side chambers contained either an unfamiliar conspecific in an overturned plastic container that had holes through which the mice on either side could sniff, but not contact, one another or an identical overturned container with no mouse [50–52]. Mice serving as social stimuli were naïve males from a different untreated litter and had been confined previously to the container so that they were habituated to the procedure and did not try to escape. The mouse being tested was allowed to freely explore the arena for 10 min. Preference towards the conspecific was measured by time spent in each chamber, and number of entries to each chamber. At the end of this trial, after a 1 min interval, during which the experimental mouse was held in the middle chamber, a second mouse was put in the side chamber that had contained the object in the SP test and the SN test started. Once again the experimental mouse was allowed to freely explore the three chambers for 10 min and time spent with each stimulus mouse (familiar or novel) was scored. The sessions were filmed and preference was coded by an observer blind to the treatment condition using Ethovision software.

#### Social conditioned place preference (SCPP)

Two male mice from the same litter were housed in a standard home cage with one of two novel types of bedding, either aspen sawdust or shredded paper. Notably, pilot data on mice that did not participate in this study indicated that there was no inherent overall preference for either type of bedding but that individual mice tended to show a preference for one or the other bedding.

The social environment was determined by pretesting the mice for bedding preference, such that for each mouse the ‘social’ cue was the non-preferred bedding. The following day, the experimental mouse was isolated in a cage that contained its preferred bedding, alternating every 24 h with a “social” environment, namely housing with a cagemate from the same litter with the non-preferred bedding. Conditioning was carried out over 10 days, alternating daily between the social and isolated conditions. Thus, successful social conditioning would result in a change of preference. Food and water were available *ad lib*.

The conditioned preference for the two distinct types of bedding was assessed for each mouse using the aforementioned three-chambered apparatus, allowing the mouse to freely explore the box for 20 min while being filmed from above. Each side contained a cupful of one of the bedding materials spread on the floor of the chamber, counter-balancing the side among animals. Time spent in each chamber was scored via the Ethovision software by an observer blind to the treatment condition.

### Repetitive novel object contact task (RNOC) (PND 90)

This test was designed to evaluate repetitive behaviors towards a novel object [38]. Each mouse was placed in a round 40 cm diameter arena and underwent 5 min habituation on three consecutive days. One day following habituation, the mouse was placed again in the arena that now contained three novel objects, constructed from several Lego-like blocks, which were placed equidistant from one another. Each object was constructed from 2 to 3 blocks about 5–6 square centimeters in area, differing in shape. The mouse was allowed to freely explore the arena and the objects for 5 min. Time spent engaging in exploration of the objects was analyzed via Ethovision software. Object positions were counterbalanced and after every trial, the arena and objects were cleaned.

### Object recognition (PND 90)

In order to rule out the possibility that exposed mice had a more global impairment that affected their recognition memory, object recognition was examined. The ability of animals to discriminate between objects requires recognition of the previously explored object, and detection of the differences between the objects [53]. The object recognition task was performed in a plastic circular 53 cm in diameter apparatus. Mice were habituated to the arena and to two identical Lego-like objects that were placed in it for 3 consecutive days, for 15 min each time. On the fourth day, one object was replaced by a novel, different shape, Lego-like object that was located in the exact same place. Object recognition was tested in a 10 min trial. Mouse performance was scored by an experimenter blind to the manipulation.

### Statistical analysis

Data were analyzed using parametric analysis of variance (ANOVA). For the reflexes, a repeated measure ANOVA was conducted for the effect of treatment (between subjects) and day (within subjects) for days 6–12. For the tests conducted in adults, ANOVA was conducted for the effect of prenatal treatment for each of the dependent variables, followed by post hoc comparisons between the CPF groups and each of the two control groups. Effect size is shown as partial eta squared.

## Results

### Early development and neuromotor behavior (PND’s 6–12)

#### Body weight

A two-way ANOVA with repeated measures was conducted for the effect of Age (PND 6–12) and Treatment (NT-No Treatment, Vehicle, 2.5 mg/kg CPF, or 5 mg/kg CPF) on body weight revealed the expected significant effect of Age, F(6150) = 213.60, *p* < .000001, indicating growth of the pups, no main effect of Treatment F(3,25) = 1.41, n.s., but a significant interaction between Age and Treatment, F(18,150) = 2.72, *p* < .005). The mean weight of the groups was compared using the Least Significant Difference post hoc test in order to see if CPF exposure significantly affected weight gain. The LSD test revealed that on PND 10–12, the group exposed to 5 mg/kg *in utero* had a higher average weight than the group that had been exposed to 2.5 mg/kg CPF *in utero* and on PND 11–12, the 5 mg/kg group weighed more than the group treated with oil vehicle. However, on the days when reflexes were impaired (see below) there were no group differences in weight (Table 1).

**Table 1:** Mean (SEM) daily body weight (gr) of pups during reflex testing

DAY	6	7	8	9	10	11	12
No Treatment	4.13 (0.19)	4.29 (0.61)	4.71 (0.63)	5.11 (0.59)	5.56 (0.58)	5.98 (0.70)	6.47 (1.0)
Vehicle	3.6 (0.49)	3.85 (0.70)	4.25 (0.69)	4.8 (0.76)	5.19 (0.74)	5.48 (0.79)	5.89 (0.87)
CPF 2.5 mg/kg	3.13 (0.29)	3.63 (0.46)	4.06 (0.74)	4.58 (1.19)	4.98 (1.31)	5.36 (1.27)	5.76 (1.39)
CPF 5 mg/kg	3.53 (0.62)	4.01 (0.76)	4.56 (0.74)	5.18 (0.80)	6.02 (0.77)	6.48 (0.87)	6.95 (0.93)

#### Righting reflex

**Fig. 1:**
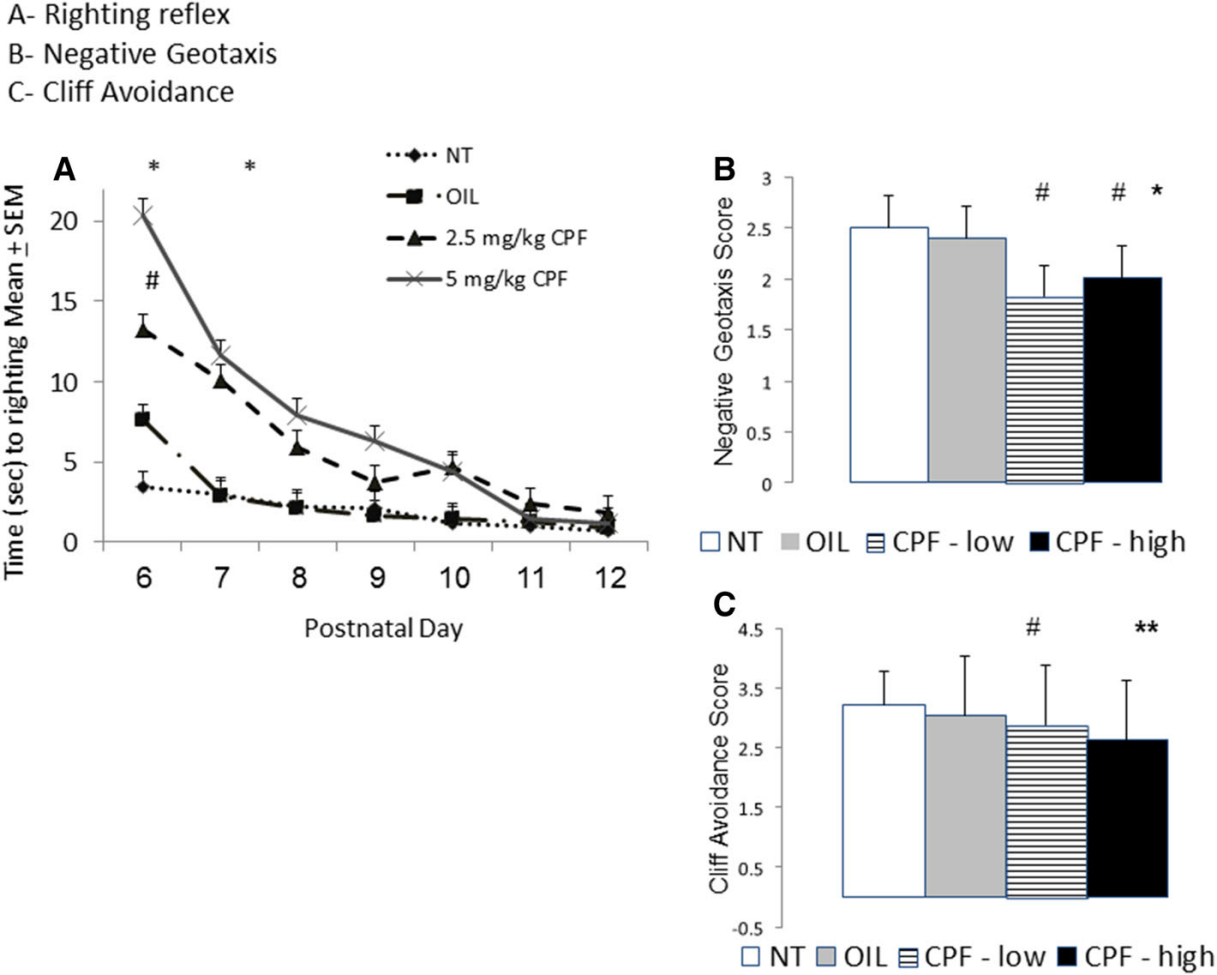
Postnatal righting reflex (**a**), negative geotaxis reflex (**b**) and cliff avoidance reflex (**c**) in pups whose dams were given no treatment (NT), or gavaged with Vehicle, or 2.5 or 5 mg/kg CPF in oil once daily on GD12-15. The number of pups per groups was NT = 8, VEHICLE = 7, 2.5 mg CPF = 6, 5 mg/kg = 8. Statistically significant differences are marked as follows: **a** * *p* < .05 compared to NT and VEHICLE. # *p* < .05 compared to NT and VEHICLE for 5 mg/kg CPF. *P* < .05 5 mg/kg CPF vs VEHICLE. **b** *Lines* represent significant differences *p* < .05. **c** *p < .05 vs each of the other groups. #*p* < .05 compared to NT

A two-way ANOVA for the effects of Treatment x Age (repeated measure) was conducted on the daily score for each reflex. There was a significant main effect of Treatment, F(3,25) = 9.63, *p* < .001, η^2^p = .54, and the expected effect of Age, indicating the reduction in righting time as the mice matured, F(6150) = 57.87, *p* < .00001, η^2^p = .46. Notably, the CPF treatment delayed the development of the righting reflex as seen by the significant interaction between Age and Treatment, F(18,150) = 3.22, *p* < .0005, η^2^ p = .28. The mice treated with 2.5 mg/kg or 5 mg/kg CPF had slower righting reflexes than the Vehicle and NT control groups on PND 6 and 7. The higher dose of CPF also led to a slower righting reflex compared to both control groups on PND 8 and compared to the Vehicle-treated control group on PND 9 (Fig. 1a).

#### Negative geotaxis

The two-way ANOVA revealed a significant effect of Treatment, F(3,25) = 14.91, *p* < .0001, η^2^p = .63 and the expected maturation effect reflected by the significant effect of Age, F(6, 150) = 28.07, *p* < .000001, η^2^p = .53. The interaction was not significant, F(18,150) = 1.52, n.s. Post hoc least significant difference (LSD) comparisons confirmed that both CPF-treated groups had lower average scores on the negative geotaxis reflex than the Vehicle and NT control groups. The difference between the two doses of CPF was not statistically significant, (*p* = .06), but suggested a trend to a greater effect with the higher dose.

#### Cliff avoidance

A main effect of Treatment was found, F(3,25) = 13.52, *p* < .00005, η^2^p = .64, as well as the expected effect of Age, F(6, 150) = 16.72, *p* < .000001, η^2^p = .40. The interaction was not significant, F(18,150) = 1.21, n.s. Post hoc LSD comparisons confirmed that during the testing period the average score was lower in the 5 mg/kg CPF-treated mice compared to the NT, Vehicle and 2.5 mg/kg CPF groups. The mice pretreated with 2.5 mg/kg CPF showed lower scores compared to the NT group, but not compared to the vehicle control group (*p* = .07), although there was a clear trend suggesting an impairment (Fig. 1c).

### Social behavior in adults (PND 90)

#### Social preference and social novelty

**Fig. 2:**
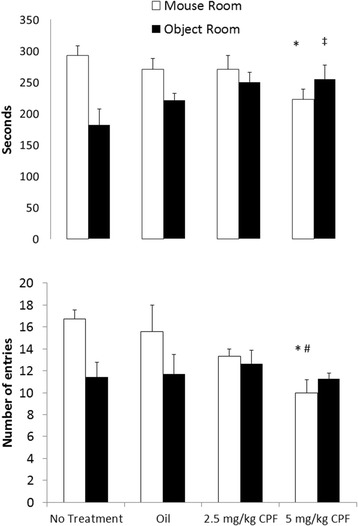
Social preference Mean + SEM time in seconds (*top*) and number of entries (*bottom*) into the side containing the novel mouse compared to the side with the inanimate object. * *p* < .05 compared to the NT and Vehicle groups. The number of mice per group is NT, Vehicle and 5 mg/kg = 7 and 2.5 mg/kg = 3

In the SP task, a significant interaction between the time spent in each chamber (mouse vs object) and Treatment (NT, Vehicle, 2.5 or 5 mg/kg CPF) was found, F(3,20) = 3.36, *p* < .05, η^2^p = .33. Post hoc comparisons revealed that mice treated with 5 mg/kg of CPF spent less time with another conspecific compared to Vehicle and NT groups, *p* < .05 (Fig. 2a). We also found a significant interaction between the number of entries to the Side and Treatment, F(3,20) = 5.36, *p* < .01, η^2^p = .45. Post hoc comparisons revealed significant differences in the number of entries to the mouse room between the high CPF group and the Vehicle and NT groups. There was no significant difference in the amount of time spent in the object chamber between the Vehicle and either the high CPF, F(1,20) = 0.88, n.s., or low CPF group, F(1,20) = .37 (Fig. 2b).

In the SN task, however, there was no significant main effect of Side (familiar vs novel mouse), although there was the expected trend to prefer the novel mouse over the familiar mouse F(1,16) = 3.36, *p* = 0.08. There was no effect of Treatment (NT, Vehicle, 2.5 or 5.0 mg/kg CPF), F(3,16) = 1.78, n.s. The interaction between Side and Treatment was not significant, F(3,16) = 1.23, n.s. (Table 2), suggesting that although there was a tendency to prefer the novel mouse, this tendency was not significantly affected by the prenatal CPF treatment.

**Table 2:** Mean + SEM time in seconds spent in chamber containing the familiar mouse and the novel mouse in the Social Novelty Test

Group/Time	Familiar mouse	Novel mouse
No Treatment	207.923 (9.96)	310.306 (9.16)
Vehicle	203.628 (9.61)	327.156 (8.20)
CPF 2.5 mg/kg	257.02 (6.18)	254.333 (4.97)
CPF 5 mg/kg	241.18 (18.68)	243.237 (20.36)

### Social conditioned place preference

**Fig. 3:**
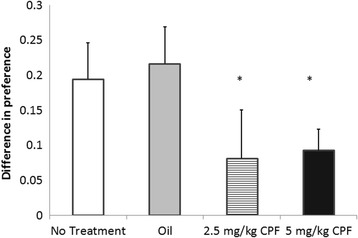
Socially conditioned change in preference for a previously non-preferred bedding (Mean + SEM). * *p* < .05 compared to the Vehicle group. Number of mice per group was NT = 8; Vehicle = 7; 2.5 mg/kg = 5; 5 mg/kg = 12

Since we used a biased SCPP paradigm, our dependent variable was the difference between the percent of time spent with the initially non-preferred bedding prior to and following social conditioning. The change in preference was compared among the three treatment groups and was found to be affected by CPF, F (3, 28) = 2.94, *p* = .05. η^2^p = .24. Post hoc comparisons revealed that the Vehicle control group showed a higher change in conditioned social bedding preference than each of the CPF exposed groups (*p* < .05), and the difference between the NT and the CPF groups showed a strong trend towards statistical significance (*p* = .08 for 2.5 mg/kg and *p* = .053 for 5 mg/kg) (Fig. 3).

### Repetitive novel object contact task (RNOC) (PND 90)

**Fig. 4:**
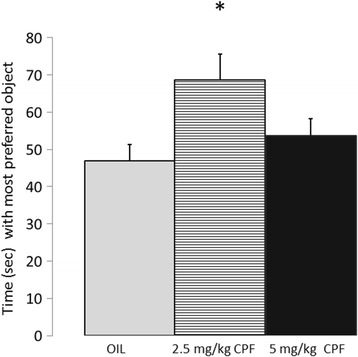
Mean + SEM preference for a single object in the RNOC test. * *p* < .05 compared to Vehicle. The number of adult mice tested was: Vehicle = 7, 2.5 mg/kg CPF = 8 and 5 mg/kg CPF =10

A main effect for CPF dose was found, for the ratio of time the mouse spent with its preferred object in relation to total time exploring the 3 objects F(2,22) = 3.48, *p* < .05, η^2^p = .24. Post hoc comparisons revealed a significant difference between mice exposed to 2.5 mg/kg and the vehicle group, *p* < .5. (Fig. 4).

No difference was found between groups for the total amount of time spent within 1 cm of the objects, F (2,22) = 1.57, n.s. or the time spent with the mouse’s preferred object, the object with which the mouse spent the most time, F (2,22) = 1.59, n.s. suggesting that the enhanced object preference in the CPF group was not due to a motor or sensory deficit. Of the three objects, all were preferred by some of the mice, with one of the objects being preferred by 12 of the 25 mice, including about half the mice in each group. The other two objects were favored by 6 and 7 mice from the sample of 25.

### Object recognition (PND 90)

Time spent in proximity of 2 cm to the object was computed for every object. The ratio between the time spent with the novel object relative to total time spent with the familiar and novel objects was calculated: F(2,15) = 0.305, ns. Differences between mice treated with vehicle (mean = 0.62, SEM = 0.017), 2.5 mg/kg CPF (Mean = 0.695, SEM = 0.022) and 5 mg/kg CPF (Mean = 0.613, SEM = 0.063) treated mice were not found. There were no differences between groups in the total amount of time spent with objects: F(2,15) = 1.06, ns, suggesting that gestational CPF did not affect recognition or memory ability.

### Maternal care (PND 5)

The latency to retrieve each pup was compared by 2-way ANOVA with pup as a repeated measure. The latency for one dam for pup 2 (Vehicle group) was missing, so that the group average was substituted for that mouse. A significant effect of PUP was found, indicating that the first pup was retrieved more slowly than the other two pups, F (2, 28 = 7.46, *p* < .01, η^2^p = .35. There was no significant difference between groups in the maternal retrieval task and no interaction between group and pup (F < 1 in both analyses), suggesting that maternal care was not impaired in dams that received CPF by gavage (Table 3).

**Table 3:** Maternal pup retrieval test

	Retrieval pup1	Retrieval pup2	Retrieval pup3
Vehicle (*N* = 4)	79.09 (11.95)	10.59 (0.99)	13.99 (1.68)
CPF 2.5 mg/kg (*N* = 9)	83.81 (8.6)	29.45 (5.01)	24.76 (4.21)
CPF 5 mg/kg (*N* = 4)	80.12 (9.03)	35.25 (8.45)	18.13 (4.78)

Time (sec) required to retrieve 3 pups in dams treated with CPF or vehicle on gestational days 12–15. Data are indicated as Mean + SEM

## Discussion

Taken together, our results indicate that gestational CPF interferes with early neuromotor development, causes deficits in social behavior and increases restricted interest in adulthood. Notably, although long-term effects of early exposure to CPF or chlorpyrifos oxon on USV emission and social interactions have been reported [31], this is the first study to demonstrate a consistent pattern of both spontaneous and learned social behavior as well as restricted interest in adults that had been exposed to CPF during gestation. Moreover, the observed anomalies do not appear to be related to maternal care or to a memory deficit.

In the early stages of postnatal development, gestational exposure to CPF resulted in delayed development of neonatal reflexes; however, the observed anomalies in all three neonatal reflexes were transient, as was the weight difference which was not evident during the period in which the deficits in reflexes were apparent. At 12 days of age, mice that were exposed to CPF performed as well as mice that were not exposed, suggesting that these basic motor functions were not impaired at the time the social and RNOC behaviors were tested in adults. Our results are in line with results from Engel et al., [17], who found a similar deficit in neuromotor development in newborn infants, who displayed abnormal reflexes after *in utero* and early postnatal exposure to organophosphates [54]. Infants at high risk or who were later diagnosed with autism spectrum disorders (ASD) showed more deficits in reflexes and spontaneous motor movement during childhood than typically developing children [55]. Although delays in the development of reflexes are by no means specific, the pattern of developmental and adult deficits that emerged from this study warrants further investigation of the effects of gestational exposure to CPF on social functioning and restrictive interest at different ages.

In adults, we found that mice that were treated with 5 mg/kg CPF did not show preference towards a conspecific in the SP task, which assesses sociability in mice, and has been validated in genotypes related to autism in humans such as neural cell adhesion molecule null mice, neuroglinin-4 null mice and fragile X mental retardation protein deficient mice [56–58]. In contrast to our findings following gestational CPF, postnatal exposure to CPF on PND 11–14, did not impair social preference SP [45]. Together, these data suggest that different behaviors are affected by CPF exposure in different critical periods from gestation throughout the preweanling period [29, 59, 60] and that the effects of prenatal exposure to CPF do not completely overlap with those of postnatal exposure. Critical periods of sensitivity to the detrimental effects of developmental exposure to CPF on social behavior will require further investigation in a single study that compares different treatment regimens. Although the mice that had been exposed to CPF did not show a deficit in the SN task, this could be related to the fact that they did not show a preference for the supposedly familiar mouse on the SP task which preceded the SN task. The lack of preference for the mouse over the inanimate object precludes the ability to subsequently recognize this same mouse as “familiar” in the SN test. Thus, the absence of a CPF effect in the SN task is consistent with their “failure” to prefer to spend time with a conspecific over an object.

In the SCPP task, in contrast to both control groups, mice that were treated with CPF failed to show a socially-conditioned change of preference towards bedding that was paired with a social stimulus (‘social environment’). The fact that control groups did reverse their bedding preference indicates that social conditioning is a powerful reinforcer. The resistance of CPF- treated mice to preference change is reminiscent of ASD-like exaggerated need for routine. Further studies are required to determine if CPF-treated mice are able to switch their preference following conditioning for a non-social reward.

The mechanism of the long-term effects of gestational CPF remain undetermined. CPF administration during development alters the levels of monoamine systems and was found to modify the expression of numerous genes, many of which are involved in neuronal and glial development and other regulated processes unrelated to the AChE inhibition [61]. Another likely mechanism could involve alterations in the neurohypophysis hormones, oxytocin and vasopressin, which are known to affect social behavior and repetitive or stereotyped behaviors [62, 63]. Gestational, but not postnatal CPF treatment induced a significant elevation in hypothalamic oxytocin (OT) and concomitant decrease in arginine vasopressin (AVP) in male, but not female adult mice [64]. The behavioral tests in the current study indicate that gestational CPF may lead to long-term deficits in social behavior and repetitive behavior, supporting findings linking pervasive developmental disorder symptoms in 24 and 36 month old children to gestational OP exposure [18, 19]. Further research is required to examine the biological underpinnings of these deficits.

## Conclusions

This study indicates that gestational exposure to CPF caused delayed motor development, impaired conditioned and innate social behaviors and increased restricted interest. This is the first study to report long-term effects of developmental exposure to CPF on restricted interest and social behavior in the same cohort of mice. Notably, these abnormalities are similar to those manifested in infants and toddlers whose mothers had been exposed to OP’s, such as abnormal reflexes and PDD (e.g., [12]). Since human research is limited and inevitably confounded by subjects’ personal history and the fact that subjects are exposed to a mixture of pesticides, this study provides a basis on which to pursue further exploration of the neurobiological mechanisms underlying the behavioral deficits. Several possible mechanisms are likely to be relevant [65]. A recent imaging study found an association between prenatal exposure to CPF and smaller volume of several prefrontal cortical brain areas involved in regulating emotional behavior in 7 year old children [66], suggesting that morphological studies might shed light on the observed changes in behavior. Gestational chlorpyrifos oxon at doses that inhibited AChE by approximately 85% [32], appears to mitigate some of the behavioral or structural deficits found in mice expressing low levels of reelin, suggesting that reelin might be relevant for the long-term effects of chlorpyrifos exposure [67]. While the current study does not reveal the underlying mechanism, it demarcates a multi-faceted ASD-like behavioral profile in male mice following gestational exposure to CPF. Future studies should focus on reversal of the behavioral changes and analysis of morphological changes to the brain, as well as exploring the role of sex hormones which are known to be affected by CPF [68].

## Abbreviations

AChE: Acetylcholinesterase
ANOVA: Analysis of variance
ASD: Autism spectrum disorder
CPF: Chlorpyrifos
OP: Organophosphate pesticides
PDD: Pervasive developmental disorder
PND: Postnatal day
RNOC: Repetitive novel object contact
SCPP: Social conditioned place preference
SN: Social novelty
SP: Social preference
